# Viscoelastic heating of insulated bovine intervertebral disc

**DOI:** 10.1002/jsp2.1002

**Published:** 2018-03-09

**Authors:** Harrah R. Newman, Robert D. Bowles, Mark R. Buckley

**Affiliations:** ^1^ Department of Biomedical Engineering University of Rochester Rochester New York; ^2^ Department of Bioengineering University of Utah Salt Lake City Utah

**Keywords:** energy dissipation, heat capacity, intervertebral disc, uniaxial compression, viscoelastic heating

## Abstract

Back pain is the leading cause of disability globally and the second most common cause of doctors’ visits. Despite extensive research efforts, the underlying mechanism of back pain has not been fully elucidated. The intervertebral disc (IVD) is a viscoelastic tissue that provides flexibility to the spinal column and acts as a shock absorber in the spine. When viscoelastic materials like the IVD are cyclically loaded, they dissipate energy as heat. Thus, diurnal, regular movements of the vertebral column that deform the IVD could increase disc temperature through viscoelastic heating. This temperature rise has the potential to influence cell function, drive cell death and induce nociception in innervating nociceptive neurons within the IVD. The present study was conducted to investigate the capacity of IVD to increase in temperature due to viscoelastic heating. Insulated caudal bovine IVD were subjected to physiological cyclic uniaxial compression over a range of frequencies (0.1‐15 Hz) and loading durations (1‐10 min) ex vivo*,* and the temperature rise in the tissue was recorded. According to our findings, the IVD can experience a temperature rise of up to 2.5°C under cyclic loading. Furthermore, under similar conditions, the inner nucleus pulposus exhibits more viscoelastic heating than the outer annulus fibrosis, likely due to its more viscous composition. The measured temperature rise of the disc has physiological relevance as degenerative IVD tissue has been shown to produce a sensitization of nociceptive neurons that spontaneously fire at 37°C, with a T50 response at 37.3°C and a maximum response at 38°C. Our results suggest that viscoelastic heating of IVD could interact with sensitized nociceptive neurons in the degenerative IVD to play a role in back pain.

## 1 INTRODUCTION

Intervertebral discs (IVDs) are composite, fibrocartilaginous structures that act as shock absorbers between vertebrae in the spine. The IVD consists of 2 distinct parts: the fibrocartilaginous outer annulus fibrosus (AF) and the more viscous inner nucleus pulposus (NP). Collectively, the IVD is a viscoelastic tissue.^1^, ^2^, ^3^ Viscoelastic materials possess mechanical properties that resemble both viscous fluids and elastic solids.^4^ When cyclically loaded, viscoelastic materials lose energy in the form of heat as a result of viscous dissipation. This phenomenon is known as viscoelastic heating, and can cause a time‐varying increase in tissue temperature that depends on its heat capacity, thermal conductivity, and thermal boundary conditions. Increased tissue temperature due to viscoelastic heating has previously been observed both in vivo and ex vivo in tendon, another viscoelastic connective tissue.^5^, ^6^, ^7^ For example, temperatures as high as 45°C were recorded in the superficial digital flexor tendon (SDFT) of galloping horses in vivo^7^ and the rate of temperature rise in dynamically loaded sheep plantaris tendons was found to increase with mechanical power in vitro.^6^ However, to our knowledge, viscoelastic heating has not been investigated in the IVD.

In vivo, IVD undergo cyclic multiaxial deformations,^8^ including axial compression, as a result of regular movements, physical activities, and exposure to vibrations. Individuals operating heavy machinery, such as construction equipment, experience full body vibrations up to and exceeding 15 Hz.^9^, ^10^, ^11^ Over time IVD can degrade as a result of age^12^ or injury, resulting in reduced function and significant pain.^1^ Heating in the IVD is of particular interest because recent investigations have shown that degenerative human IVD is capable of sensitizing nociceptive neurons to thermal stimuli.^13^, ^14^ As a result, nociceptive neurons begin to fire at 37°C, have a T50 of 37.3°C and see a maximal firing response by 38°C. As a result, relatively small changes in temperature in the disc could drive nociception neuron firing in the degenerative IVD. Moreover, super‐physiological temperatures could affect cell health, induce cell death,^15^ and impact homeostasis of the extracellular matrix by altering enzyme activity. Hence, viscoelastic heating could conceivably play an important role in IVD pain and degeneration.

The primary objective of the current study was to investigate the capacity of IVD, both as a whole and divided into its individual constituents (AF and NP), to increase in temperature as a result of viscoelastic heating when cyclically loaded over a range of frequencies and test durations. To this end, we subjected well‐insulated, intact bovine IVD, as well as separated AF and NP, to cyclic uniaxial compression ex vivo. We hypothesized that tests of higher frequency and longer test duration would result in greater heating and temperature rise in the IVD than those of lower frequency and shorter test duration. Additionally, we hypothesized that the temperature increase associated with viscoelastic heating would be higher in the NP than in the AF due to its viscous nature. Finally, we hypothesized that the measured temperature rise of the IVD would closely correlate with both the energy dissipated and the theoretical maximum temperature increase in the case of perfect insulation.

## 2 MATERIALS AND METHODS

### 2.1 Sample preparation

**Figure 1:**
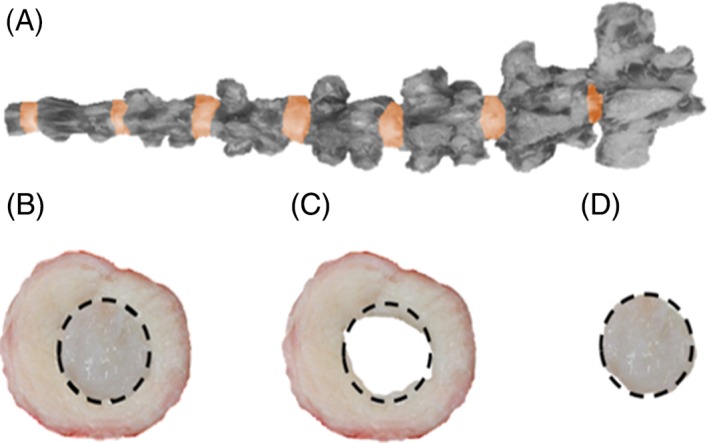
(A) Photograph of intact bovine tail with bone segments recolored in gray scale to highlight IVD (see online version for color). (B) Intact IVD after dissection. (C) Outer annulus fibrosus and (D) inner NP after isolation

Healthy bovine tails were acquired from a local abattoir and caudal IVD were carefully dissected from between the vertebrae (Figure 1A) and stored at −20°C for a maximum of 5 days. Prior to testing, IVDs were thawed and kept hydrated in phosphate‐buffered saline (PBS). For *control tests* and *IVD heating experiments*, IVDs were kept intact and were measured to have diameters of 23.2 ± 2.50 mm (mean ± SD) and thicknesses of 6.74 ± 0.80 mm. For *regional heating experiments*, IVDs were separated into the AF and NP prior to testing (Figure 1B‐D).

### 2.2 Experimental setup

**Figure 2:**
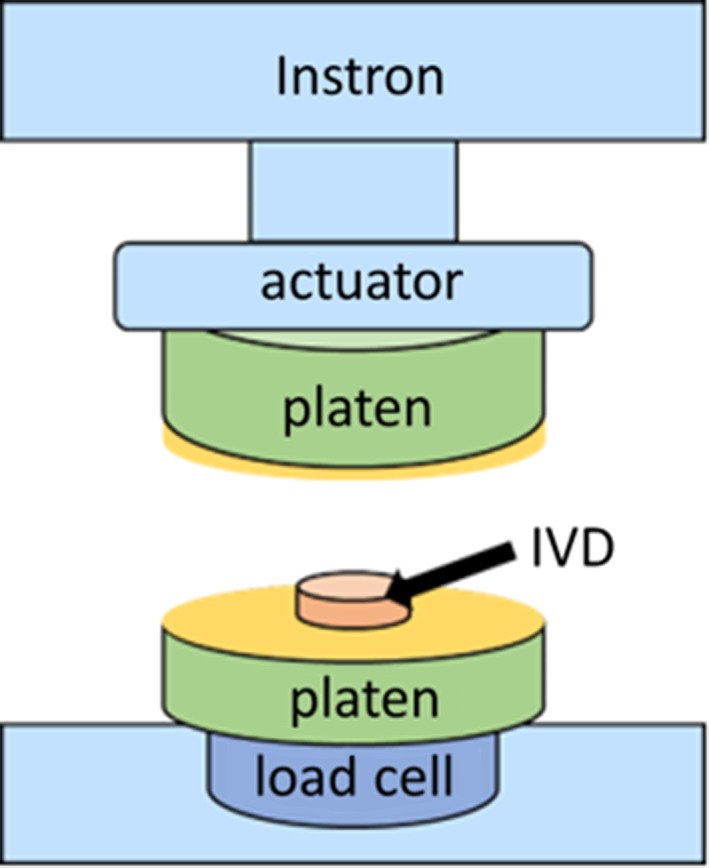
Schematic of mechanical testing equipment. An Instron Electropuls E10000 was used to cyclically compress insulated IVD (insulation not shown)

Thawed and insulated IVD, AF or NP were equilibrated to room temperature and placed between 2 flat, custom fabricated platens in an Instron Electropuls E10000 materials testing device (Instron, Norwood, Massachusetts; Figure 2). The top platen was attached to the actuator arm, and the bottom platen was attached to a 1‐kN load cell. The actuator arm was displaced such that the tested specimen was subjected to uniaxial compression along the cranial‐caudal direction. The insulation methods and loading protocols are described in later sections.

Before and after testing, the temperature of the center of the specimen (or, in the case of AF only tests, the center of the circular cross section of the AF) was measured using a sharp k‐type thermocouple. The temperature rise, Δ*T* of the specimen was taken to be the difference between the temperature of the specimen after testing and the temperature of the IVD prior to testing. The thermocouple was removed from the tissue during testing, as friction between the tissue and thermocouple could cause the disc to heat independent of internal mechanisms.

### 2.3 Insulation tests

To assess the capacity of the IVD to rise in temperature due to viscoelastic heating, it was necessary to insulate the specimens such that the heat generated within the tissue was not immediately transferred to the surroundings. In vivo, despite temperature regulation by nearby vasculature, some degree of insulation due to surrounding tissues (eg, endplates and spinal ligaments) is likely to exist as well. Thus, several methods of insulation were considered and characterized.

Preliminary trials were conducted with bare, uninsulated discs; however, this methodology was determined to be inadequate due to nonphysiological evaporative cooling. To avoid evaporative cooling, additional tests were performed with the IVD in a PBS bath. This form of insulation was also deemed inadequate since the temperature of both the tested specimen and the PBS bath itself increased. The temperature rise of the PBS bath occurred even in the absence of a specimen between the platens, and this fluid temperature rise—presumably due to viscous dissipation around the moving platen—was difficult to decouple from the independent temperature rise of the tissue due to viscoelastic heating.

Note that controlling the PBS bath temperature has the potential to counteract platen‐associated heating. However, heat from a heating element used to control bath temperature could be difficult to delineate from viscoelastic heating. Moreover, no temperature control strategy can prevent local (or temporary) increases in temperature. Thus, despite the use of temperature control, motion of the top plate is likely to cause a local temperature elevation in the fluid immediately surrounding the disc. This fluid heating could increase the temperature of the disc and could be misinterpreted as an effect of viscoelastic heating. Importantly, the possible presence of a temperature rise due to IVD‐independent mechanisms has the potential to confound the interpretation of measurements of disc temperature. Therefore, we did not attempt to use a heated/temperature controlled PBS bath in our experiments.

**Figure 3:**
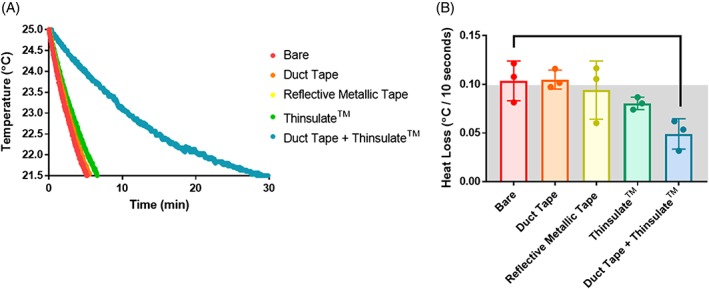
(A) Heat loss of IVD for each form of insulation for cooling from 25°C to 21.5°C. Each curve represents the average temperature decay for a given form of insulation (*n* = 3). Insulation with duct tape + Thinsulate led to the slowest heat loss. (B) Heat loss per 10 s time interval for each form of insulation (*n* = 3). Only insulation with duct tape + Thinsulate was associated with a significantly lower rate of heat loss compared to bare disc. The gray box represents the accepted error range of the thermocouple used (±0.1°C)

Due to the shortcomings of testing bare specimens and testing in a fluid bath, we determined that it was necessary to wrap the disc within an insulating material. Several insulative wrappings were tested and compared: a reflective metallic tape (to prevent heat loss due to radiation), a layer of woven fabric surrounded by a thin sheet of polyethylene (duct tape), a compact set of thin insulating fabric sheets (Thinsulate) and a layered insulation of duct tape + Thinsulate. For these experiments, an IVD (*n* = 3/insulation method) was placed in a PBS bath heated to approximately 30°C. After thermal equilibration, the IVD was removed from the bath and wrapped in insulation. A sharp k‐type thermocouple was then secured in the center of the disc to continuously record the internal IVD temperature. The IVD was left at room temperature (~20°C) until it cooled to 21.5°C. According to a one‐way analysis of variance (ANOVA) with Bonferroni post hoc analysis (*α* = .05), the average rate of heat loss was only significantly lower than bare IVD in duct tape + Thinsulate, where it took nearly 30 min for the temperature to decrease by just 3.5°C (Figure 3). Thus, we selected this method of insulation for use in all mechanical tests. The total thickness of the duct tape + Thinsulate insulation was 1 to 1.4 mm under a preload of 25 N (see “cyclic experiments”), as compared with the thickness of the IVD (6.74 ± 0.80 mm). Due to the nonnegligible thickness of the insulation, the target applied strain amplitude (~20%) may differ slightly from the actual imposed tissue‐scale strain amplitude.

### 2.4 Control tests

Several control sets were conducted to ensure that mechanical testing did not induce a temperature rise independent of tissue viscoelastic heating. First, to ensure that the insulation itself did not exhibit measurable viscoelastic heating, a stack of Thinsulate sheets wrapped in duct tape with the same thickness as the insulated IVD (~8 mm) was loaded into the Instron and compressed cyclically to a strain of −20% of the uncompressed disc height for 10 min at 15 Hz (*n* = 3). Next, to ensure that the mechanical testing system itself did not measurably heat the disc, insulated IVDs (*n* = 5) were adhered to the top platen with tape and cyclically displaced *without contacting the bottom platen* (such that the IVD was not compressed) for 10 min at 15 Hz with an amplitude of 20% of the IVD thickness. Finally, to confirm that *static* loading and deformation of the disc—which should lead to negligible energy dissipation—do not heat the disc through a different mechanism, IVDs (*n* = 5) were compressed to a static load of 25 N and the central temperature was recorded before loading and 10 min after loading.

Additional control tests were performed to evaluate the effect of thermocouple puncture on disc heating. The thermocouple used in our study had a diameter of 1.23 mm. A previous study showed that puncture of caudal bovine discs from needles with a diameter of 0.82 mm does not impact overall mechanics in bovine caudal IVD.^16^ Furthermore, it was observed that a needle diameter: disc height ratio exceeding 0.4 is necessary to impact disc mechanics.^17^ The thermocouple diameter: disc height ratio in this study was approximately 0.18, below the 0.4 threshold. Nevertheless, experiments were conducted to confirm that thermocouple puncture did not affect viscoelastic temperature rise.^31^, ^32^ To this end, specimens with and without thermocouple puncture (*n* = 4 per group) were cyclically compressed to −20% strain for 1 min at 15 Hz. The predicted maximum temperature rise (explained later) was then calculated and compared between the punctured and unpunctured discs.

Finally, 2 sets of experiments were conducted to ensure the repeatability of testing at different frequencies. In the first experiment discs were (1) compressed cyclically to −20% strain for 1 min at 1 Hz; (2) allowed to rest 25 to 30 min; (3) tested for 1 min at 15 Hz; (4) allowed to rest for another 25 to 30 min; and (5) tested again for 1 min at 1 Hz. In the second set of experiments, the same procedure was followed but with an initial and final test at 15 Hz and middle test at 1 Hz.

### 2.5 IVD and regional heating experiments

To assess the capacity of IVD, AF, and NP to rise in temperature due to viscoelastic heating, thermally equilibrated specimens insulated with duct tape + Thinsulate were loaded into the Instron and preloaded to −20 N (where the negative sign indicates a compressive force) to ensure full contact between the platens and the insulated IVD. This preload ensured that the top platen visually remained in contact with the insulated IVD throughout the time course of the experiment. As soon as the preload was reached, the tissue was subjected to cyclic uniaxial compression with a triangular waveform to a peak strain of −20% of the uncompressed disc height with a frequency of 0.1, 1, 3, 5, 10, or 15 Hz and a duration of 1, 5, or 10 min, depending on the trial. The temperature of the IVD was assessed before and after the test as described earlier, after which the IVD was removed from the insulative wrapping and immersed in PBS for at least 2 h before the next test.

For each test duration, including 1, 5, and 10 min tests, 5 intact IVD specimens were tested across all frequencies, for a total of 15 intact IVD tested. Testing was conducted such that for each test duration group, every disc was tested once before repeating the test on the same disc with a different frequency. The order of test frequency was randomized as an extra precaution to ensure that increased heating was not an effect of the number of tests the disc previously experienced.

Another 5 discs were separated into their respective AF and NP constituents, which were then individually tested at a single frequency and test duration (15 Hz and 5 min) according to the same protocol used for intact IVD.

### 2.6 Calculation of dissipated energy and the maximum possible temperature change

The load cell force (*F*) and actuator displacement (*∆L*) recorded by the Instron and the dimensions of each IVDs were used to calculate the dissipated energy and maximum possible temperature change of each tested specimen. First, the axial engineering stress (*σ*_*zz*_) was determined from *F* and the initial disc diameter (*d*) according to

(1) $$\sigma_{\mathrm{zz}}=\frac{F}{A}=\frac{F}{\pi {\left(\frac{d}{2}\right)}^{2}}.$$

The axial infinitesimal strain (*ε*_*zz*_) was computed from *∆L* and the initial thickness of the disc (*L*_0_) according to

(2) $$\varepsilon_{\mathrm{zz}}=\frac{∆L}{L_{0}}.$$

**Figure 4:**
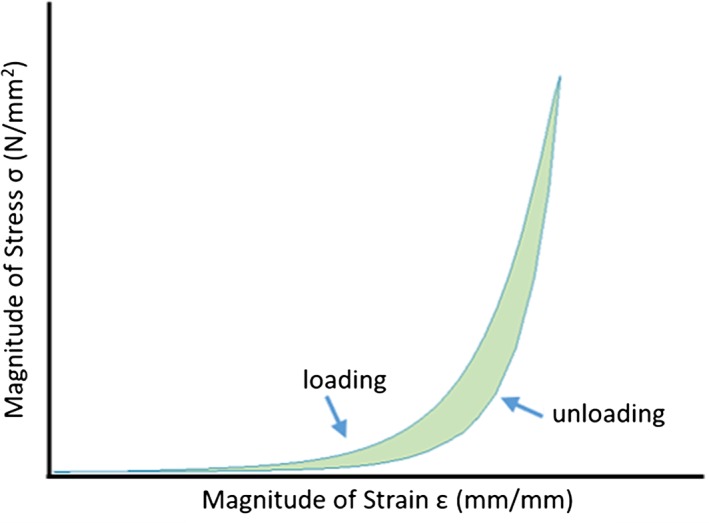
Representative (magnitude of) stress versus (magnitude of) strain plot for a single cycle of a cyclically compressed, intact IVD. The area in between the curves (green) is equal to the energy dissipated during this cycle per unit volume. Note that since the IVD undergoes compression, the actual stress and strain are both negative

For each cycle, the stress‐strain curve exhibits hysteresis characteristic of a viscoelastic material. The area inside this curve is equal to the energy dissipated per tissue volume during cycle *i* ($W_{i}^{\text{dissipated}}$; Figure 4). This parameter was calculated for each cycle *i* using the MATLAB trapezoidal numerical integration function “trapz” to compute the area within the hysteresis loop. The dissipated energies $W_{i}^{\text{dissipated}}$ in each cycle were then summed together to obtain the total energy dissipation per volume over the course of the entire test ($W_{\text{test}}^{\text{dissipated}}$):

(3) $$W_{\text{test}}^{\text{dissipated}}=\sum_{i}W_{i}^{\text{dissipated}}.$$

If we assume that all dissipated energy is dissipated as heat, then $W_{\text{test}}^{\text{dissipated}}$ is equal to the total heat energy generated in the tissue. If we further assume that the tissue is perfectly insulated (such that no heat transfer occurs with the surroundings), using the definition of specific heat capacity *c*, the change in temperature *∆T*_max_ of the tissue in terms of $W_{\text{test}}^{\text{dissipated}}$, *c* and tissue density *ρ* is given by

(4) $$\Delta T_{\max}^{\text{predicted}}=\frac{W_{\text{test}}^{\text{dissipated}}}{\mathrm{\rho c}}.$$

For IVD, we take the density *ρ* to be 1100 kg/m^3^ and the specific heat capacity *c* to be 3568 J kg^−1^°C^−1.^ 18 Note that since this calculation assumes that (1) all dissipated energy is dissipated as heat; and (2) the IVD is perfectly insulated, $\Delta T_{\max}^{\text{predicted}}$ represents the maximum theoretical temperature increase from viscoelastic energy dissipation over the course of the experiment.

### 2.7 Expected relationship between specimen size and temperature rise at equilibrium

In an ideal situation featuring perfect insulation, for a given rate of energy dissipation per volume, viscoelastic heating and the resulting temperature increase of the disc increase with time and are independent of specimen dimensions (Equations 3 and 4). However, the insulation of our experimental system is imperfect. In this case, not only will heating be slower than in the ideal scenario, but in addition, the temperature will reach an asymptote at long times (when the rate of heat generation is balanced by the rate of heat loss) that depends on the specimen size. Specifically, according to heat transfer theory, the temperature rise at equilibrium is given by

(5) $${∆T}_{\text{equilibrium}}=\frac{HL^{2}}{2\kappa },$$

where *κ* is thermal conductivity (constant), *H* is the energy dissipated per second per volume, *L* is the disc height (assuming that the disc is cylindrical), and *∆T*_equilibrium_ is the expected asymptotic temperature increase. Note that in this calculation, we are assuming that the disc is thin such that heat loss is predominantly axial (rather than radial).

### 2.8 Statistical analysis

For *IVD heating experiments*, Δ*T* was compared across frequency and test duration using a 2‐way ANOVA with Bonferroni post hoc analysis. According to a power analysis of main effects performed using the pwr2 package in the statistical software R (https://www.r-project.org/), the design of this study yielded power of 0.99 to detect a 0.25°C difference in Δ*T* across frequencies and a power of 0.91 to detect a 0.25°C difference in Δ*T* across durations. The strength of the correlations between Δ*T* and $\Delta T_{\max}^{\text{predicted}}$ and the correlations between *∆T*_equilibrium_ and disc height *L*^2^ were assessed for each loading duration by computing the coefficient of determination, *R* ^2^. For *regional heating experiments*, Δ*T* was compared across regions (AF vs NP) for a single frequency and duration (15 Hz and 5 min) using a paired Student’s *t*‐test. For all analyses, the threshold for significance was set to *α* = .05.

## 3 RESULTS

### 3.1 Control tests

**Figure 5:**
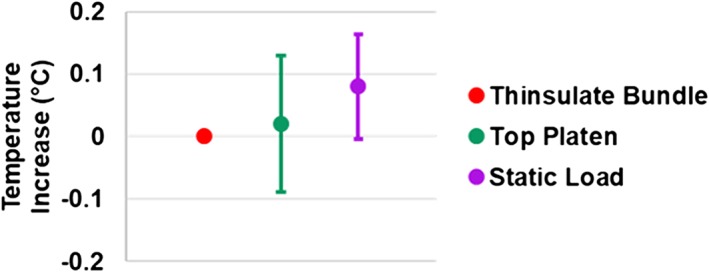
Temperature increase for 3 control tests (*n* = 3‐5/test): (1) testing of insulation only; (2) application of cyclic displacement on the materials testing system without allowing the specimen to contact the bottom platen (ie, without applying load); (3) application of a static load. In all cases, the measured heating is within the error range of the thermocouple (±0.1°C)

**Figure 6:**
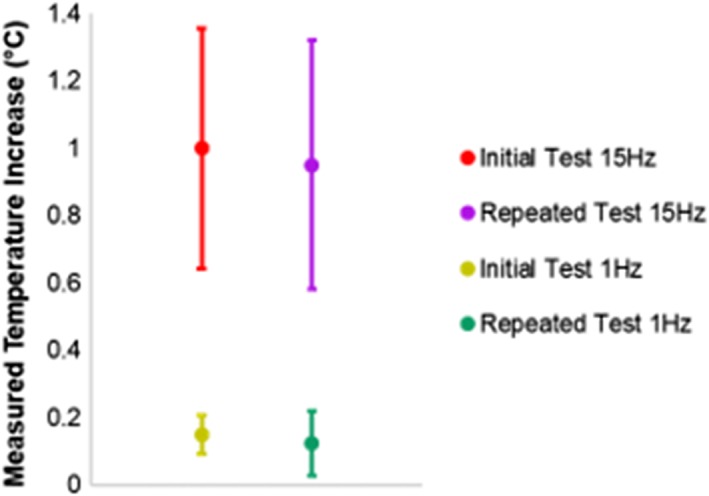
Two sets of experiments were performed to investigate the repeatability of assessments of ΔT (*n* = 4 per experiment). An initial test was conducted for 1 min at either 15 or 1 Hz. The disc was allowed a 30 min rest, then subjected to a second test at a different frequency (either 1 or 15 Hz) before a second 30 min rest period. Finally, the original test was repeated and the temperature rise was compared between the initial and repeated test. The temperature rise was not significantly different between the initial and repeated tests for both 15 Hz (*P* = .18) and 1 Hz (*P* = .72)

Control tests of Thinsulate + duct tape only, insulated discs dynamically displaced without contacting the bottom platen, and statically loaded discs all yielded negligible heating within the resolution of the thermocouple (±0.1°C) (Figure 5). Additionally, there was no significant difference in maximum theoretical temperature increase between punctured and unpunctured discs (*P* > .44), confirming that thermocouple puncture did not impact IVD heating. Finally, tests conducted at the same frequency before and after an intermediate test yielded no significant difference in temperature rise, indicating that test order did not impact viscoelastic heating (Figure 6).

### 3.2 IVD heating experiments

**Figure 7:**
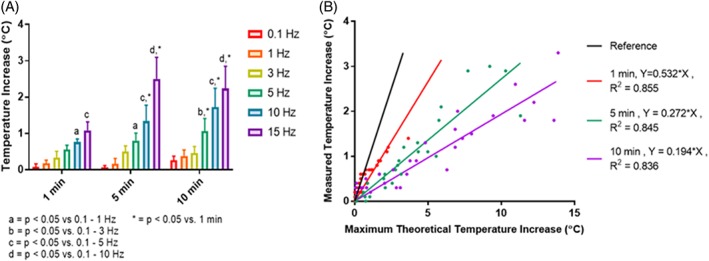
(A) Temperature rise for cyclic compression of intact IVD across loading frequencies for durations of 1, 5, and 10 min. Temperature rise was generally greater at higher frequencies and longer loading durations (up to 5 min). (b) Comparison of the maximum theoretical temperature increase (based on the total energy dissipated) with the measured temperature increase. According to linear regression analysis of data from all frequencies, there was a strong correlation (*R* ^2^ > 0.8) between these parameters for all loading durations. However, the slope of each linear fit was less than 1 (see solid black reference line) and decreased with loading duration, indicating that the maximum theoretical temperature increase was generally higher than the measured temperature increase, and more so for linger loading durations

Under cyclic loading, the IVD increased in temperature by as much as 2.5°C. The measured temperature increase (Δ*T*) generally increased with both frequency and loading duration (Figure 7A). For example, for a 1‐min loading duration, Δ*T* was significantly greater at 15 Hz than at 0.1 to 1 Hz while for 5 and 10 min loading durations, Δ*T* was significantly greater at 15 Hz than at 0.1 to 10 Hz. Moreover, for 10 and 15 Hz loading frequencies, Δ*T* was significantly greater for 5 and 10 min of loading than for 1 min of loading. However, the effect of loading duration disappeared for long loading durations, as no significant differences were found between loading for 5 and 10 min.

The measured temperature rise Δ*T* and the maximum theoretical temperature rise $\Delta T_{\max}^{\text{predicted}}$ (calculated based on the energy dissipated per cycle measured from the stress‐strain curve) were strongly correlated for all loading durations (*R* ^2^ > 0.836). Moreover, the slope of the linear regression line (reflecting the multiplicative factor relating Δ*T* to $\Delta T_{\max}^{\text{predicted}}$) increased with increasing load duration (Figure 7B).

**Figure 8:**
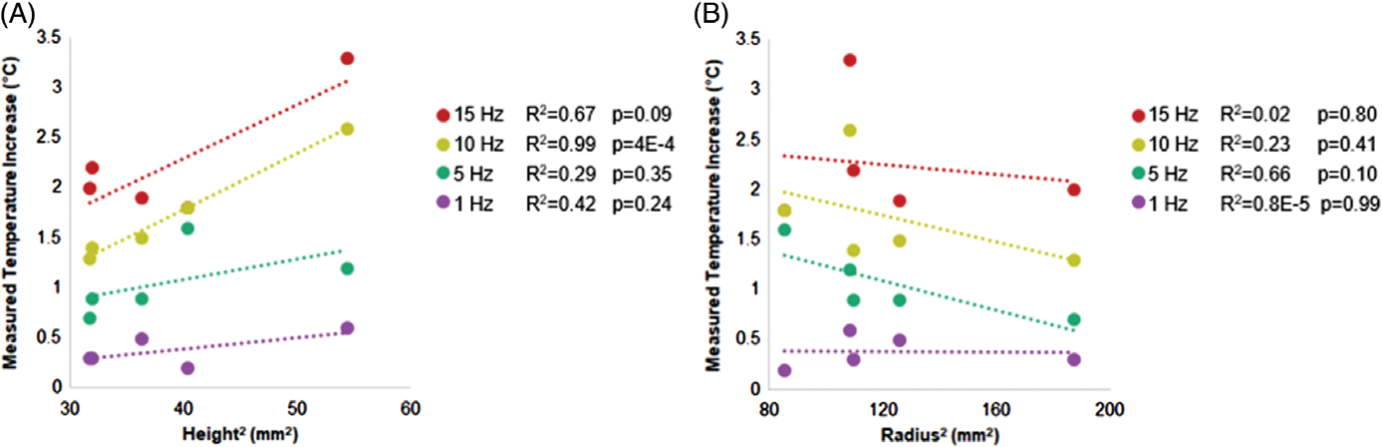
(A) The correlation between measured temperature increase and disc height squared was moderate (0.25 < *R* ^2^ < 0.75) to strong (*R* ^2^ > 0.75) for all 10 min tests (1‐15 Hz) and significant (*P* = .0004) for 10 min tests at 10 Hz. (B) The correlation between measured temperature increase and disc radius squared was weak (*R* ^2^ < 0.25) to moderate (0.25 < *R* ^2^ < 0.75) for all 10 min tests (1‐15 Hz), but was not significant at any frequency (*P* > .1)

Since the measured value of *∆T* does not increase significantly between 5 and 10 min of loading, we take this value of *∆T* to be the asymptotic temperature rise *∆T*_equilibrium_. The disc features a large surface area on the top and bottom compared to the area along the circumference, which facilitates axial heat loss over radial heat loss. Axial heat loss would imply that *∆T*_equilibrium_ would scale with *L*^2^ (disc height squared), whereas with radial heat loss, *∆T*_equilibrium_ would be expected to scale with *r*^2^ (radius squared). The experimental data yielded a stronger correlation between *∆T*_equilibrium_ and *L*^2^ (Figure 8A) than between *∆T*_equilibrium_ and *r*^2^ (Figure 8B), as anticipated. For example, for loading at 10 Hz for 10 min, the correlation between *∆T*_equilibrium_ and *L*^2^ was significant (*P* = .0004) and strong (*R*^2^ = 0.99) while the correlation between *∆T*_equilibrium_ and *r*^2^ was not significant (*P* = .41) and moderate (*R*^2^ = 0.23). Moreover, *∆T*_equilibrium_ generally decreased with increasing *r*^2^, which would not be the case if heat were dissipated radially. Therefore, our data support axial heat loss as predicted by heat transfer theory (Equation 5). The measured temperature increase at equilibrium was strongly to moderately correlated with disc height squared at the higher frequencies of 15 and 10 Hz (Figure 8A). Due to their long duration, these high frequency tests are most likely to reach the asymptotic temperature *∆T*_equilibrium_. Therefore, the measured temperature increase and the square of the height correlate more closely in these tests than in tests conducted at the lower frequencies of 5 and 1 Hz, where correlations were moderate (Figure 8A).

### 3.3 Regional heating experiments

**Figure 9:**
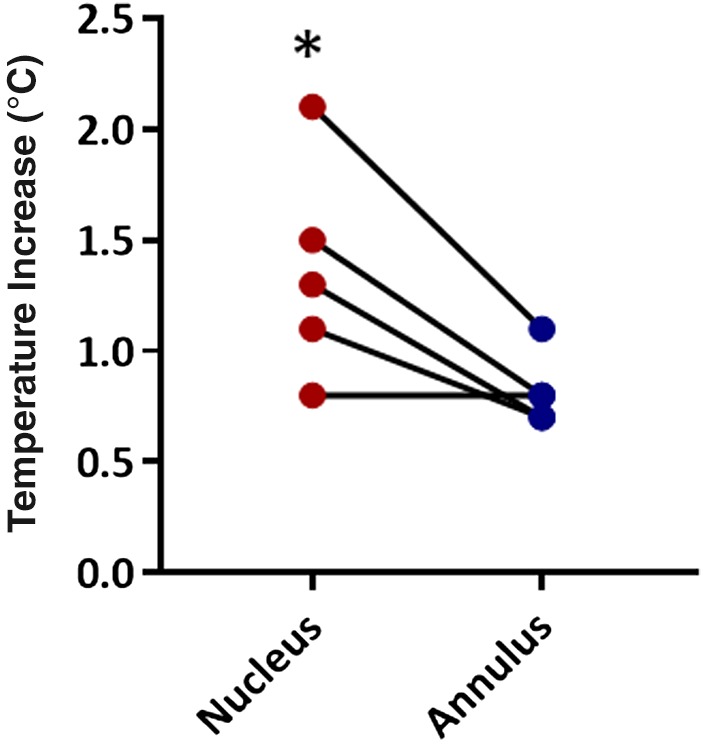
Independent heating of isolated, cyclically loaded nuclei (red) and annuli (blue) (*n* = 5/group). The temperature rise in the nucleus was significantly higher, and there was greater heating in the nucleus in all but one pair. * indicates *P* < .05 versus annulus

Δ*T* was significantly higher in the NP than in the AF (Figure 9). In fact, in all but one disc separated into the AF and NP, Δ*T* was greater in the nucleus than in the annulus.

## 4 DISCUSSION

To our knowledge, this is the first study to investigate the capacity of the IVD to rise in temperature as a result of viscoelastic dissipation. As hypothesized, we found that under certain conditions, viscous energy loss during cyclic loading is sufficient to measurably increase the temperature of the IVD. Additionally, Δ*T* increased with both frequency and loading duration, attaining values higher than 2°C with 15 Hz loading for at least 5 min. Moreover, the measured temperature increase Δ*T* correlated strongly with $\Delta T_{\max}^{\text{predicted}}$. Finally, separating the AF and NP revealed that the NP heated more under identical conditions.

Several separate findings in this study strongly suggest that the measured temperature increase Δ*T* is due predominantly to viscoelastic heating of IVD. First, control tests were performed to (1) assess viscoelastic heating of the insulation material used to prevent heat loss from the IVD; (2) assess heating of the IVD due to the materials testing system itself; and (3) assess heating of the IVD due to static (elastic) deformation. Each of these tests yielded negligible heating. In addition, we found that Δ*T* was strongly correlated with $\Delta T_{\max}^{\text{predicted}}$, the theoretical IVD temperature rise calculated from the measured energy dissipated (based on load‐displacement data). This strong correlation between Δ*T* and a *completely independent* assessment of viscoelastic heating further indicates that viscoelastic heating is the primary cause of the measured rise in IVD temperature.

The sensitivity of Δ*T* to both loading frequency and loading duration was expected, since both parameters scale with the total number of loading cycles (*n*) and $\Delta T_{\max}^{\text{predicted}}$ scales with *n* and the energy dissipated per cycle $W_{i}^{\text{dissipated}}$ (Equations 3 and 4). In other words, a higher number of loading cycles leads to more energy dissipation, increased viscoelastic heating and greater temperature rise. Frequency could also affect Δ*T* through its influence on $W_{i}^{\text{dissipated}}$. Specifically, for sinusoidal loading at small strains, $W_{i}^{\text{dissipated}}$ is proportional to the square of the strain amplitude and the loss modulus *E*^″^, 19 which is in turn proportional to sin *δ* (where *δ* is the frequency‐dependent loss tangent equal to the phase angle between stress and strain). However, previous studies have shown that *E*^″^ and *δ* vary weakly with frequency in IVD,^20^, ^21^ suggesting that the frequency dependence of $W_{i}^{\text{dissipated}}$ is playing a limited role in the frequency dependence of Δ*T*.

Although Δ*T* was strongly correlated with $\Delta T_{\max}^{\text{predicted}}$ for all loading conditions, Δ*T* was almost always lower than $\Delta T_{\max}^{\text{predicted}}$. Moreover, the ratio of Δ*T* to $\Delta T_{\max}^{\text{predicted}}$ (ie, the slope of the linear regression modeling the relationship between Δ*T* and $\Delta T_{\max}^{\text{predicted}}$) decreased with loading duration, suggesting that $\Delta T_{\max}^{\text{predicted}}$ overestimates Δ*T* to a greater extent the longer the disc is loaded. These findings can be explained by the imperfect insulation capacity of duct tape + Thinsulate. Although the calculation of $\Delta T_{\max}^{\text{predicted}}$ assumes that the IVD is perfectly insulated, some heat loss will occur over the course of each experiment. Moreover, the extent of heat loss will increase for longer loading durations because (1) there is more time for heat transfer for occur; and (2) because as energy is dissipated and the disc temperature increases, the temperature difference between the disc and the room (which governs the rate of heat transfer) will also increase.

As anticipated, due to imperfect insulation, the viscoelastic temperature rise *∆T* equilibrated at long times. In particular, it did not increase significantly between 5 and 10 min of loading, allowing us to take the values of *∆T* at 10 min of loading to be the asymptotic temperature rise *∆T*_equilibrium_. Furthermore, our experimental data (Figure 8) confirm a direct relationship between disc size and equilibrium temperature elevation, as predicted by heat transfer theory (Equation 5). Given this relationship, we can predict the expected temperature increase in human IVD under the same test conditions we used to test the bovine IVD. If we assume that *κ* and *H* are equal in bovine and human IVD:

(6) $$\frac{{∆T}_{\text{equilibrium}}^{\text{human}}}{{∆T}_{\text{equilibrium}}^{\text{bovine}}}=\frac{L_{\text{human}}^{2}}{L_{\text{bovine}}^{2}}.$$

In this study, the average height of bovine disc (6.74 mm) and equilibrium temperature rise experienced by bovine disc for 15 Hz loading (2.24^°^C) were assessed. In the human disc, the average height is approximately 11.3 mm. 22 Thus, for a 10‐min test at 15 Hz, we anticipate that

(7) $${∆T}_{\text{human}}={∆T}_{\mathrm{cow}}\frac{L_{\text{human}}^{2}}{L_{\mathrm{cow}}^{2}}\sim \left(2.24^{{}^{\circ}}C\right)\frac{{\left(11.3\mathrm{mm}\right)}^{2}}{{\left(6.74\mathrm{mm}\right)}^{2}}\sim 6.30^{{}^{\circ}}C.$$

From these calculations, we can conclude that the expected equilibrium temperature increase in our system would be about 6°C in human IVD, as compared to about 2°C in bovine IVD.

Since the viscoelastic parameter *E*^″^ (the loss modulus) is related to the energy dissipated per cycle in a material subjected to sinusoidal loading and we have shown that the total energy dissipated in the IVD is correlated with the increase in tissue temperature, it is possible to relate this temperature rise to *E*^″^. In particular, in a linear viscoelastic material subjected to sinusoidal loading, the energy dissipated per cycle per volume is given by $W^{\text{dissipated}}=\pi \gamma_{0}^{2}E^{\prime \prime }$ where *γ*_0_ is the applied strain amplitude. Thus, the energy dissipated per volume after time *t* for loading at frequency *f* is given by $W_{\text{test}}^{\text{dissipated}}=\pi \gamma_{0}^{2}E^{\prime \prime }\text{ft}$. Assuming perfect insulation, the increase in disc temperature can then be expressed as

(8) $$\Delta T_{\max}^{\text{predicted}}=\frac{W_{\text{test}}^{\text{dissipated}}}{\mathrm{\rho c}}=\frac{\pi \gamma_{0}^{2}E^{\prime \prime }\text{ft}}{\mathrm{\rho c}}.$$

According to our findings (Figure 7), Δ*T* is an approximately linear function of $\Delta T_{\max}^{\text{predicted}}$ such that $\Delta T=\mathrm{C\Delta}T_{\max}^{\text{predicted}}$ where *C* is an experimentally determined parameter. In the case of perfect insulation, *C* = 1. But practically, *C* is less than 1 and depends on *t* due to the time‐dependent escape of heat from the disc. For example, *C* = 0.558 for 1 min of loading, *C* = 0.289 for 5 min of loading and *C* = 0.207 for 10 min of loading (Figure 7). Note that in our experiments, we cannot measure *E*^″^ since we did not apply sinusoidal deformation. However, our data nevertheless demonstrate how Δ*T* can be predicted from knowledge of *E*^″^, *t*, *ρ*, *c*,and *f* (Equation 8).

Our data demonstrate that while both the NP and AF exhibit viscoelastic heating, the NP heats to a greater extent than the surrounding AF when independently compressed. This finding is consistent with a previous study reporting that de‐nucleated IVD exhibit a reduction in loss modulus *E*^″^, property that (as described earlier) dictates the ability of a material to dissipate energy under cyclic loading.^20^ One source of viscous dissipation in cyclically compressed biological tissues is fluid‐solid friction as water is forcibly squeezed out. Hence, viscoelastic heating (perhaps more appropriately described in this case as “poroelastic heating”) is expected to increase with increasing water content.

Due to its higher concentration of negatively charged glycosaminoglycans, the NP has a higher water content and higher osmotic pressure than the AF.^23^, ^24^ Thus, the increased viscous heating observed in the nucleus could be due to its distinct composition. Our data imply that cells in both the annulus and nucleus are likely to be affected by viscoelastic heating. But since greater temperature rise is observed in the NP, cells in this region may be more susceptible to impaired function or cell death as a result of viscoelastic heating. It is important to note that our independent tests of AF and NP do not fully recapitulate the loading conditions that these tissue constituents experience when the IVD is intact, as the pressurization of the nucleus under compression imparts hoop stresses onto the AF that will be absent when the AF is removed.^25^, ^26^ However, since heat is easily conducted between the AF and NP in intact IVD, testing the AF and NP separately was necessary to compare their individual contributions to IVD viscoelastic heating.

Note that both poroelastic (flow‐dependent) and solid matrix viscoelastic (flow‐independent) mechanisms are likely to contribute to energy dissipation (and, consequently to heating) in the IVD. However, poroelastic effects and solid matrix viscoelastic effects may dominate at different frequencies. In isotropic and homogeneous poroelastic materials, the timescale of poroelastic relaxation for loading in an unconfined compression geometry is equal to the gel diffusion time given by $t_{\mathrm{gel}}\sim \frac{a^{2}}{H_{A}k}$, where *H*_A_ is the aggregate modulus of the material, *k* is the hydraulic permeability and *a* is the specimen radius. Taking *a* to be 0.012 m based on the measured dimensions of the tested discs and estimating *H*_*A*_ and *k* to be 0.6 MPa and 0.2 2 × 10^−15^ m^4^ N^−1^ s^−1^ based on previous studies of the AF, *t*_gel_~10^5^ min.^27^ This analysis is a very course approximation of the intact disc due to its heterogeneous composition and anisotropic structure (leading to complex matrix deformations, pressure distributions and fluid flow patterns). Nevertheless, this calculation suggests that poroelastic effects should persist even at very slow (long) timescales.

On the other hand, the timescale of solid matrix viscoelastic dissipation is expected to be shorter since this effect is caused by rapid processes including molecular relaxation,^19^ fiber sliding and bond breaking/reformation.^28^ Furthermore, at very high frequencies (much greater than $\frac{1}{t_{\mathrm{gel}}}$), load induced‐fluid flow within a poroelastic tissue may be neglected and the tissue may be treated as an incompressible material.^29^ Thus, we might anticipate that flow‐dependent effects will dominate at low frequencies while flow‐independent effects will dominate at high frequencies. However, it is important to note that there is strong evidence that across at least some of the frequencies investigated in the current study, both poroelastic and solid matrix viscoelastic effects contribute to dissipation and, consequently, should contribute to viscoelastic heating. In particular, a previous study compared the frequency‐dependent (ie, dissipative) mechanical response of IVD under different modes of deformation for frequencies ranging from 10^−3^ to 1 Hz and determined that while IVD stiffness is frequency‐dependent for both shear and compressive loading, it is more sensitive to frequency for compressive loading.^21^ Shear loading involves minimal flow‐inducing volume change and, hence, mostly probes the flow‐independent dissipative properties of a tissue. Thus, the frequency dependence of the mechanical response of IVD under shear loading suggests a solid matrix viscoelastic contribution to dissipation. In contrast, uniaxial compression involves substantial flow‐inducing volume change and probes the flow‐dependent dissipative properties of a tissue. The stronger frequency dependence of the mechanical response of IVD under compressive loading suggests a poroelastic contribution to dissipation as well (at least for frequencies between 10^−3^ and 1 Hz).

It is important to note that our results do not imply that external heating or cooling of the back (as are commonly used to treat back pain) will contribute to or help treat heat‐induced disc degeneration or disc pain. In particular, the skin and intervening muscle are both thick and highly vascularized, which aids in temperature regulation and inhibits heat transfer between the IVD and a heating or cooling pad outside of the body.

This study was not without limitations. First, testing was conducted at room temperature, not body temperature, and it is conceivable that the specific heat capacity of IVD may be temperature‐dependent. While we are unaware of any studies assessing how the specific heat capacity of IVD changes with temperature, temperature‐dependent changes in the specific heat of collagen gels have been investigated.^30^ At room temperature (296 K), the specific heat capacity of a collagen gel is approximately 1.96 *J*/*gK* and at body temperature (310 K), the specific heat capacity is about 2.02 *J*/*gK*. Assuming that the thermal properties of IVD are similar to collagen gels, according to our model (Equation 4), this increase in heat capacity of the IVD at body temperature should cause an associated decrease in temperature rise of only 3% (ie, a temperature rise of 1.94 at 37°C instead of 2°C at room temperature). Importantly, even if the specific heat capacity of the IVD is more sensitive to temperature than this calculation suggests, *changes* in temperature associated with viscoelastic heating should not strongly depend on the initial temperature of the IVD.

Second, while the duct tape + Thinsulate combination insulated the disc such that viscoelastic heating measurably increased IVD temperature, it is not clear how these experimental conditions compare with in vivo conditions. In the body, the endplates and soft tissues surrounding the IVD may provide some degree of insulation. However, nearby vasculature could help regulate the temperature of the IVD, inhibiting the effects of viscoelastic heating. Nevertheless, in galloping horses, a substantial temperature rise was observed in both the central and peripheral SDFT, suggesting that in vivo temperature regulation of connective tissues is imperfect.^7^ But tests were performed to ensure the puncture did not have significant effects on heating.

In this study, viscoelastic heating was investigated across a range of frequencies and durations for cyclic deformation to −20% strain. While the strain magnitude applied to IVD was higher than the levels of nominal strain (ie, endplate‐endplate strain) generally observed in vivo, it was within the range of local strains that occur in human discs in experimentally simulated physiological conditions.^33^ The frequency range (0.1‐15 Hz) encompassed both slow movements (0.1 Hz), ambulation (~1 Hz), and high frequency vibrations experienced while using heavy equipment (3‐15 Hz). Just as we found increased viscoelastic heating at higher frequencies, exposure of tractor, bus and heavy equipment drivers to high frequency vibrations is associated with increased risk of back pain.^9^, ^10^, ^11^, ^34^

An important advancement enabled by this study is the establishment of an in vitro experimental system that may be used to study viscoelastic heating of the IVD under well controlled conditions. In ongoing and future studies, we are using this system to investigate the influence of strain amplitude and disc degeneration on viscoelastic heating in human IVD.

Our data suggest that temperature rises >2°C are theoretically possible in IVD due to viscoelastic heating. Since it has been demonstrated that degenerative IVD can sensitize nociceptive neurons to thermal stimuli and produce a thermal dynamic firing range of 37 to 38°C, an increase in temperature above resting body temperature in the ranges demonstrated in this study could produce nociceptive signaling in the IVD and directly contribute to back pain.^13^, ^14^ Another mechanism through which viscoelastic heating of the IVD could contribute to disc degeneration is by driving apoptosis in IVD cells or otherwise inhibiting IVD cell function, as high temperatures are associated with reduced cell viability in other connective tissues.^15^ Finally, it is possible that increased disc temperature due to viscoelastic heating could disturb extracellular matrix homeostasis by speeding up the reaction kinetics of matrix metalloproteinases or other degradative enzymes. Overall, we have demonstrated a potential contributor to degenerative disc disease and back pain that has not been previously considered. Future work will characterize its role in degenerative disc tissue and investigate its role to drive nociceptive neuron firing and IVD cell function.
